# Supplementary material for: Characterising lithium-ion electrolytes via operando Raman microspectroscopy
Source: Nat Commun. 2021 Jun 30;12:4053. doi: 10.1038/s41467-021-24297-0 (PMC8245635; doi:10.1038/s41467-021-24297-0)
Supplement: Supplementary file 1 — Supplementary Information [file 41467_2021_24297_MOESM1_ESM.pdf]

Supplmentary Information

Characterising lithium-ion electrolytes via operando Raman microspectroscopy

Jack Fawdon[a], Johannes Ihli[a,b], Fabio La Mantia[c] and Mauro Pasta[a,*]

[a]Department of Materials, University of Oxford, Parks Rd, Oxford, OX1 3PH, UK
[b]Paul Scherrer Institut, 5232 Villigen, Switzerland
[c]Universtät Bremen, Energiespeicher-und Energiewandlersysteme, Bibliotechkstraße 1, Bremen, 28359, Germany
[*]mauro.pasta@materials.ox.ac.uk

Supplementary Method 1. Densitometry

Preparing concentrations on a gravimetric scale is more accurate than volumetric, as one uses an analytical balance instead of relying on the accuracy a volumetric flask. To accurately convert to a volumetric scale (molality to molarity), high precision 5-digit density measurements were run using an Anton Paar DMA 4100 density meter, located in an Argon-filled glovebox. Each measurement was temperature controlled at 20 °C, because this was the operation temperature of the Raman microscope. The density meter was rinsed with isopropanol (>=99.9%, HPLC grade, Fisher Chemical) at least three times and dried in ambient argon between measurements. The density curve is presented in Supplementary Figure 1a.

$$c_{molarity} = \frac{n_{mols}\rho}{m_{total}} \tag{1}$$

Supplementary Table 1: *Molality to Molarity*

Molality / mol kg^{-1}	Molarity / mol dm^{-3}
0.1	0.101
0.25	0.245
0.5	0.492
1	0.947
1.25	1.161
1.5	1.368
2	1.759
3	2.442

Supplementary Method 2. Partial Molar Volumes

The partial molar volumes of the salt (V_s) and the solvent (V_0) were calculated from the density and its dependence on electrolyte concentration using equations[1]:

$$V_s = \frac{M_s - \frac{\mathrm{d}\rho}{\mathrm{d}c}}{\rho - c\frac{\mathrm{d}\rho}{\mathrm{d}c}} \tag{2}$$

$$V_0 = \frac{M_0}{\rho - c\frac{\mathrm{d}\rho}{\mathrm{d}c}} \tag{3}$$

Where M_0 and M_s were the molar masses of the electrolyte (222.3 $gmol^{-1}$) and the salt (187.1 $gmol^{-1}$) respectively. Using the density curve data:

$$V_s = \frac{M_s - \rho_1 - 2\rho_2 c}{\rho_0 - \rho_2 c^2} \tag{4}$$

$$V_0 = \frac{M_0}{\rho_0 - \rho_2 c^2} \tag{5}$$

Supplementary Figure 1b and c illustrates the relationship between partial molar volumes and concentration of LiFSI in G4.

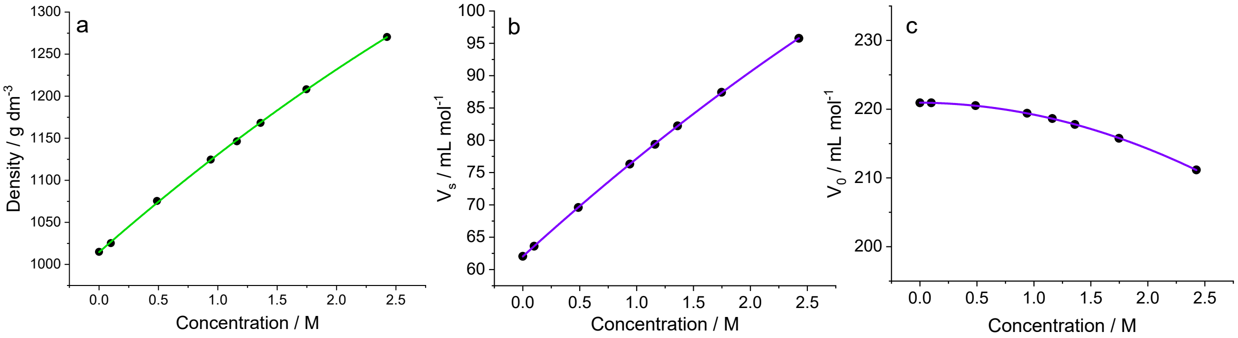

Supplementary Figure 1: *a) Density curve of LiFSI in G4 at 20 ^oC b) Partial molar volume of the salt at 20 ^oC c) Partial molar volume of the solvent at 20 ^oC*

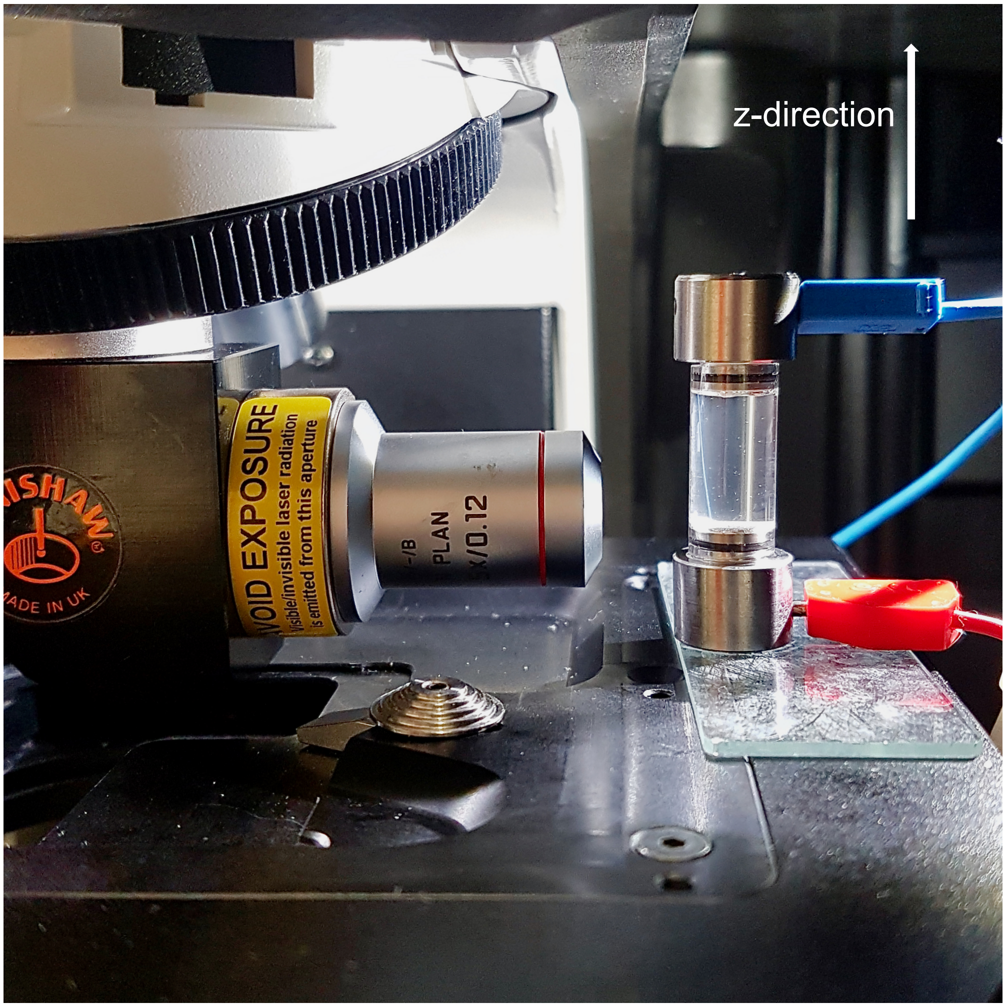

Supplementary Figure 2: *Experimental Setup: Optical cell, with two lithium electrodes, 1.5 cm apart. Stripping occurs at the bottom and plating at the top to avoid natural convection. The cell is scanned "side-on", utilising a 90° mirror, with the stage moving in the z-direction. The objective used had 5x magnification.*

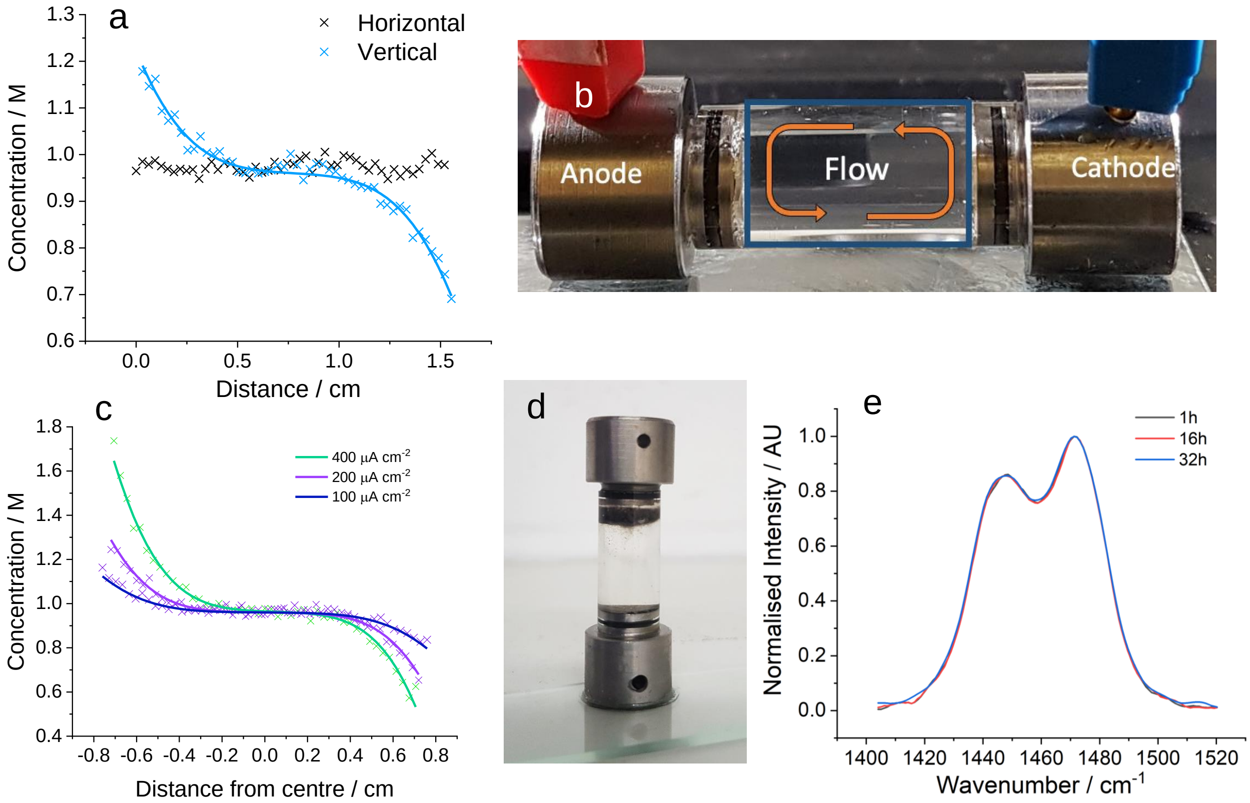

Supplementary Figure 3: *a) Concentration gradient comparison between the cells placed vertically and horizontally, after current was applied for 12 h at 100 μA cm^{-2}. If placed horizontal, the gradient suffers from natural convection due to the density gradient. b) Diagram showing annular flow of the electrolyte if the cell was placed horizontally during the experiment. Density gradients that occur perpendicular to the concentration gradient induce natural convection. c) Concentration gradient comparison between cells using currents of 100, 200 and 400 μA cm^{-2}; dendrites grew visibly at 400 μA cm^{-2}d) Example of mossy dendrite formation after applying 400 μA cm^{-2} over 48 h e) Raman spectra of 1471 cm^{-1} peak, illustrating the -CH_2 bending/scissoring not shifting over each line scan, indicating the lack of solvent decomposition.*

Supplementary Note 1. Fitting Equation

The concentration profile across the cell can be described by the solution to the diffusion equation[2] in the context of a symmetric cell:

$$\frac{\partial c_s(z,t)}{\partial t} = D_{app}\frac{\partial^2 c_s(z,t)}{\partial z^2} \tag{6}$$

The boundary conditions used are:

$$c_s(z,0) = c_s^* \tag{7}$$

$$\lim_{z\to+\infty} c_s(z,t) = c_s^* \tag{8}$$

$$D_{app}\left(1 - \frac{\partial \ln c_0}{\partial \ln c_s}\right)\left[\frac{\partial c_s(z,t)}{\partial z}\right]_{z=0} = \frac{J(1-t_+^0)}{nF} \tag{9}$$

Where c_s is the concentration of the electrolyte or salt, s, t is time, z is distance between the electrodes, c_s^* is the initial concentration, D_{app} is the "apparent" Fickian diffusion coefficient, J is the applied current density I/A, and n is the number of moles. Equation 7 states the concentration before the experiment is run. Equation 8 states the concentration will equal the initial concentration at increasing distances from the electrode surface. Equation 9 is derived from the constitutive law for cation flux:

$$N_+(t,z) = -D_{app}\left(1 - \frac{\partial \ln c_0}{\partial \ln c_s}\right)\left[\frac{\partial c_s(z,t)}{\partial z}\right] + \frac{J(z)(t_+^0)}{nF} \tag{10}$$

Where $N_+(t,z)$ is the cation flux.

Using Laplace transform:

$$\bar{c_s}(z,s) = \frac{c_s^*}{s} + A(s)e^{-\sqrt{\frac{s}{D_{app}}}z} \tag{11}$$

Where:

$$A = \frac{J(1-t_+^0)}{nFD_{app}^{\frac{1}{2}}s^{\frac{1}{2}}}\left(1 - \frac{\partial \ln c_0}{\partial \ln c_s}\right)^{-1} \tag{12}$$

And:

$$\bar{c_s}(z,s) = L\{c_s(z,t)\} \tag{13}$$

Inversion then leads to the equation that describes the concentration at the plating side of the cell:

$$c_s(z,t) = c_s^* + \frac{J(1-t_+^0)}{nFD_{app}}\left(1-\frac{\partial \ln c_0}{\partial \ln c_s}\right)^{-1}\left\{2\left(\frac{D_{app}t}{\pi}\right)^{\frac{1}{2}}\exp\left(-\frac{z}{2(D_{app}t)^{\frac{1}{2}}}\right)^2 - z\,\mathrm{erfc}\left(\frac{z}{2(D_{app}t)^{\frac{1}{2}}}\right)\right\} \tag{14}$$

Adapting 14 to measure the concentration at the opposite electrode:

$$c_s(z,t) = c_s^* + \frac{J(1-t_+^0)}{nFD_{app}}\left(1-\frac{\partial \ln c_0}{\partial \ln c_s}\right)^{-1}\left\{2\left(\frac{D_{app}t}{\pi}\right)^{\frac{1}{2}}\exp\left(-\frac{-z+L}{2(D_{app}t)^{\frac{1}{2}}}\right)^2 + (-z+L)\,\mathrm{erfc}\left(\frac{-z+L}{2(D_{app}t)^{\frac{1}{2}}}\right)\right\} \tag{15}$$

Where L is the distance between the electrodes.

Combining the two equations allows one to look at the concentration at both sides of the cell:

$$\begin{aligned} c_s(z,t) = c_s^* + \frac{J(1-t_+^0)}{nFD_{app}}\left(1-\frac{\partial \ln c_0}{\partial \ln c_s}\right)^{-1}\Bigg\{2\left(\frac{D_{app}t}{\pi}\right)^{\frac{1}{2}}\exp\left(-\frac{z}{2(D_{app}t)^{\frac{1}{2}}}\right)^2 - z\,\mathrm{erfc}\left(\frac{z}{2(D_{app}t)^{\frac{1}{2}}}\right) \\ -2\left(\frac{D_{app}t}{\pi}\right)^{\frac{1}{2}}\exp\left(-\frac{-z+L}{2(D_{app}t)^{\frac{1}{2}}}\right)^2 + (-z+L)\,\mathrm{erfc}\left(\frac{-z+L}{2(D_{app}t)^{\frac{1}{2}}}\right)\Bigg\} \end{aligned} \tag{16}$$

Equation 16 can be simplified so that only two parameters are included, a and b:

$$\begin{aligned} c_s(z,t) = c_s^* + a\Bigg\{\left(\frac{b}{\pi^{\frac{1}{2}}}\right)\exp\left(-\frac{z}{b}\right)^2 - z\,\mathrm{erfc}\left(\frac{z}{b}\right) \\ -\left(\frac{b}{\pi^{\frac{1}{2}}}\right)\exp\left(-\frac{-z+L}{b}\right)^2 + (-z+L)\,\mathrm{erfc}\left(\frac{-z+L}{b}\right)\Bigg\} \end{aligned} \tag{17}$$

Where:

$$a = \frac{J(1 - t_+^0)}{nFD_{app}} \left(1 - \frac{\partial \ln c_0}{\partial \ln c_s}\right)^{-1} \tag{18}$$

$$b = 2(D_{app}t)^{\frac{1}{2}} \tag{19}$$

For equation 17 to remain valid, $2(D_{app}t)^{\frac{1}{2}} < \frac{L}{2}$. In other words, the diffusion layer cannot extend into the centre of the cell for equation 16 to remain valid. Due to the large L that was used experimentally, the equation still held for our experiments.

Supplementary Note 2. Error Estimation Summary

Presented is a summary for each parameter's calculation and error estimation. For most of our calculations when propagating error we used the multiplication/division error propagation equation 20. We will note if we use a different equation when performing propagation with other functions.

$$\sigma_x = x\sqrt{\left(\frac{\sigma_p}{p}\right)^2+\left(\frac{\sigma_q}{q}\right)^2+\left(\frac{\sigma_r}{r}\right)^2} \tag{20}$$

D_{app} was calculated using the weighted gradient of L_d vs. $t^{1/2}$, with L_d standard error from the fitting equation 1 (main text).

t_+^0 was determined from equation 2 (main text) using the average of $dc_s/dz|_{z=0,L}$ from each fitting. t_+^0 error was propagated from the $dc_s/dz|_{z=0,L}$ standard error along with the error from the previous D_{app} calculation.

χ_M was deduced from equation 4 (main text), by plotting $\frac{F}{2RT(1-t_+^0)\eta_c}$ vs. $\ln\frac{c_{s,z=L}}{c_{s,z=0}}$. The y-axis error was calculated with t_+^0 and the x-axis had error in measuring the interfacial concentration ratio. The error in $\ln\frac{c_{s,z=L}}{c_{s,z=0}}$ (σ_x) was determined from the average interfacial concentration ratio ($c_{s,z=L}c_{s,z=0}$), which was calculated from the fitting equation (equation 1– main text), and its standard error (σ_c). This allowed the use of the error propagation equation when using natural log:

$$\sigma_x = \frac{\sigma_c}{c_{s,z=L}/c_{s,z=0}} \tag{21}$$

χ_M was subsequently calculated from linearly fitting $\frac{F}{2RT(1-t_+^0)\eta_c}$ vs. $\ln\frac{c_{s,z=L}}{c_{s,z=0}}$, weighted by x and y error.

κ and R_{ct} error was calculated from the standard deviation of four R_{bulk} measurements prior to cell polarisation. With the distance and area known very accurately, σ_κ is very small, and not noticeable in Figure 4a in the main text. A zoomed version of the κ fitting is included, highlighting the small amount of error for this measurement.

For the Stefan-Maxwell diffusion coefficients ($\mathfrak{D}$, $\mathfrak{D}_{0+}$, $\mathfrak{D}_{0-}$, $\mathfrak{D}_{-+}$), error was calculated by propagating χ_M and D_{app} error.

Supplementary Note 3. PEIS

To compare PEIS at each concentration, Supplementary Figure 4 shows the fittings of each PEIS spectrum after 4 h rest, for κ and R_{ct} measurement. The fitting used is described in the main text.

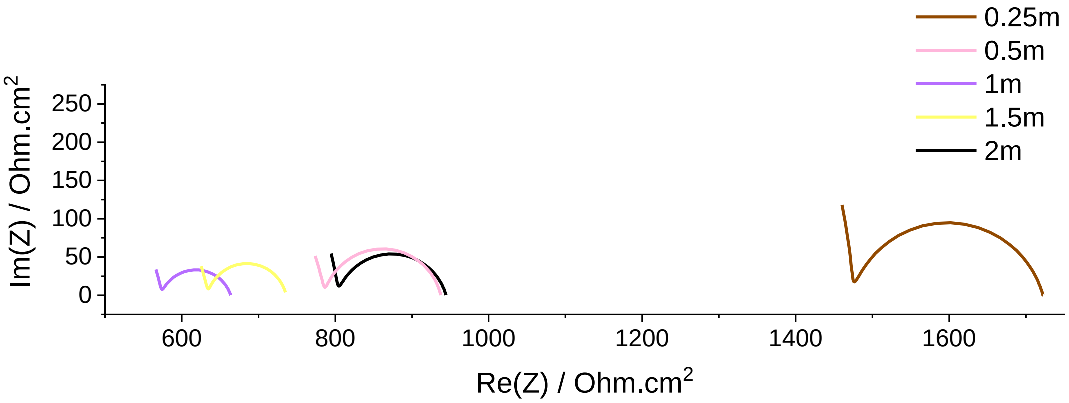

Supplementary Figure 4: *PEIS fittings of each concentration, after 4h rest, used to measure* κ *and* R_{ct}

The equation proposed by Casteel and Amis was used to fit the trend of κ vs. concentration:

$$\kappa(c) = \kappa_{max} \left(\frac{c}{c_{max}} \right)^{x_1} \exp \left(x_2 (c - c_{max})^2 - \frac{x_1}{c_{max}} (c - c_{max}) \right) \tag{22}$$

Where x_1 and x_2 are constants, c_{max} is the concentration at the maximum ionic conductivity, κ_{max}.

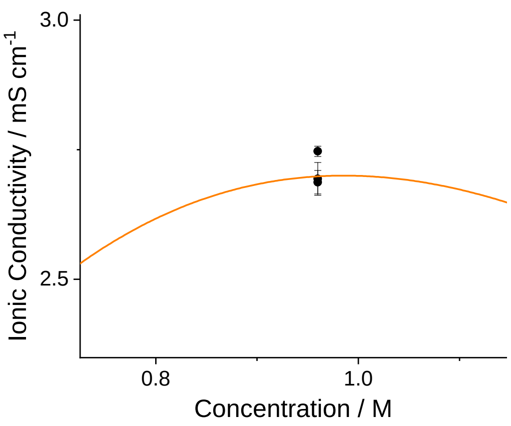

Supplementary Figure 5: *Peak of the κ curve, noting the magnitude of the error when measuring κ with PEIS*

Supplementary Note 4. Concentration Profiles

Shown in Supplementary Figure 5a is a series of selected-area Raman spectra, focusing on the normalised FSI^- 717 cm^{-1} peak. Plotted is the evolution of peak shape and intensity from the plating to the stripping electrode, highlighting changes in electrolyte environment. By monitoring the 717 cm^{-1} peak, one notices the shape and composition of the gradient that is forming. At positions closer to the stripping electrode, the shoulder peak at 720-750 cm^{-1} is more noticeable owing to the evolution of contact ion pairs (CIPs) and aggregates (AGGs), $[Li_x(G4)]_x + [FSI]_y^{(y-x)-}$.

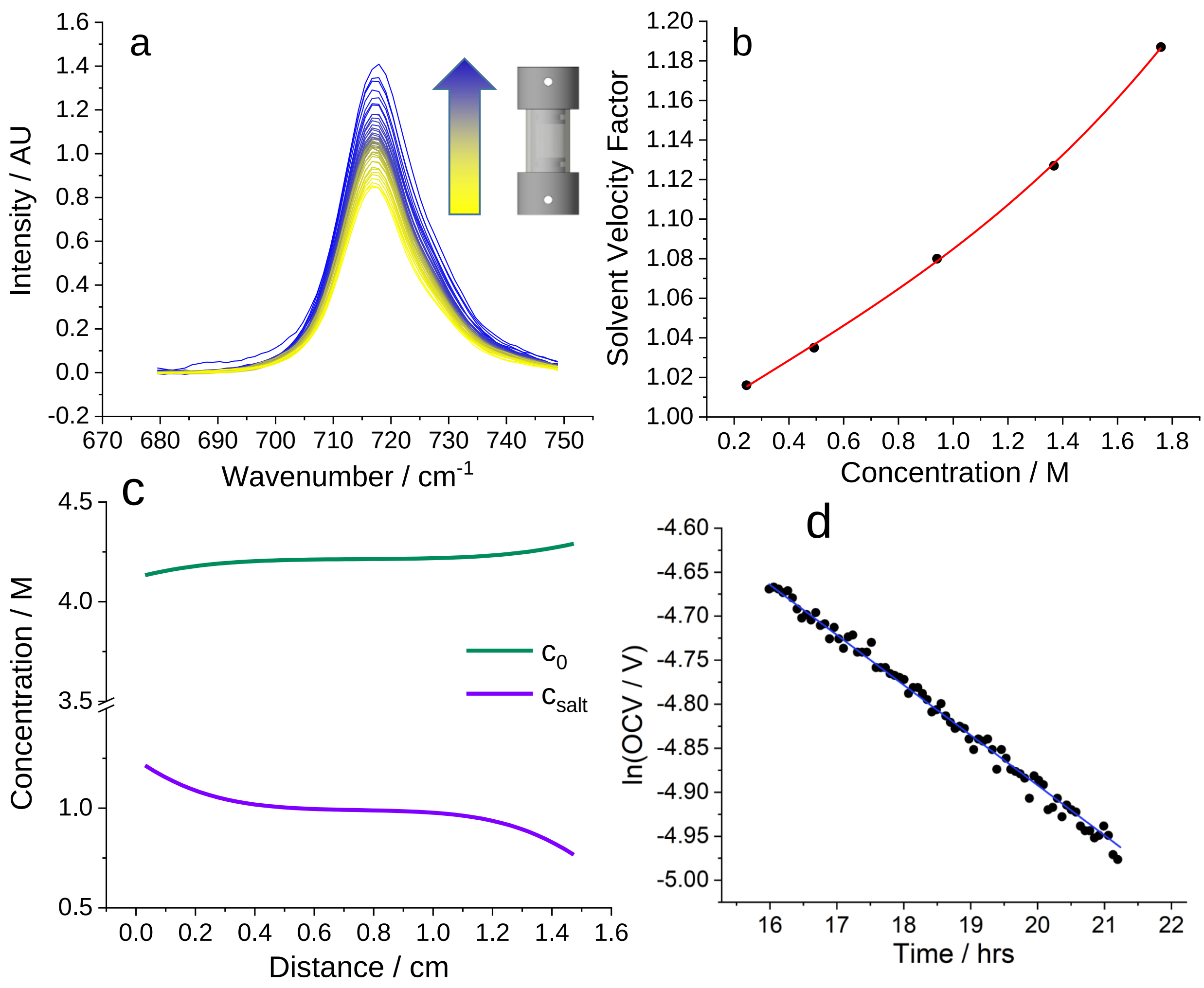

Supplementary Figure 6: *a) Raman spectra of 717 cm^{-1} peak, showing how peak area changed as one scans across the interelectrode space. b) Solvent velocity factor plotted as a function of electrolyte concentration. c) Concentration gradient fittings of the salt and solvent after current was applied for 12h at 100 μA cm^{-1}. d) ln(OCV) vs. time for D_{app} calculation using a restricted-diffusion setup.*

Supplementary Note 5. Diffusion Coefficients

To check the reliability of our D_{app} measurement, D_{app} was compared to the often-used restricted-diffusion method in the literature.[3] Using a restricted-diffusion cell and following the relaxation from an arbitrarily formed concentration gradient, the OCV decay can be followed with time, and using equation 23 below, D_{app} was calculated. The OCV curve eventually becomes linear after enough time has elapsed, and the resulting slope is proportional to D_{app}.

$$\lim_{t\to\infty} \frac{d\ln(OCV)}{dt} = -\frac{\pi^2 D_{app}}{L^2} \tag{23}$$

In this work, the same cell was used: a quartz-glass tube between two stainless steel pistons, but a shorter tube was cut so that the time for OCV relaxation was reduced. The distance between the electrodes, L, was 6.58 mm, accurately measured by the optical microscope. Crucially for this experiment L has to be small enough so that D_{app} can be measured in a reasonable time. By plotting ln(OCV) vs. time, the diffusion coefficient was calculated to be 6.98×10^{-11} m^2 s^{-1}. The results from the relaxation are shown in Supplementary Figure 6d.

Supplementary Note 6. Transference Number

Analogous to the Hittorf method,[3] we checked t_+^0 by measuring the concentration difference between each side of the cell after a molar amount of charge passed, illustrated in Supplementary Figure 7 and equation 25 below. Specifically, we measured the electrolyte molar difference between each side of the cell, which was done by integrating the measured 1D concentration gradient, and then multiplying it by the cross-sectional area of the cell. This assumes the uniform concentration in the x- and y-directions of the cell.

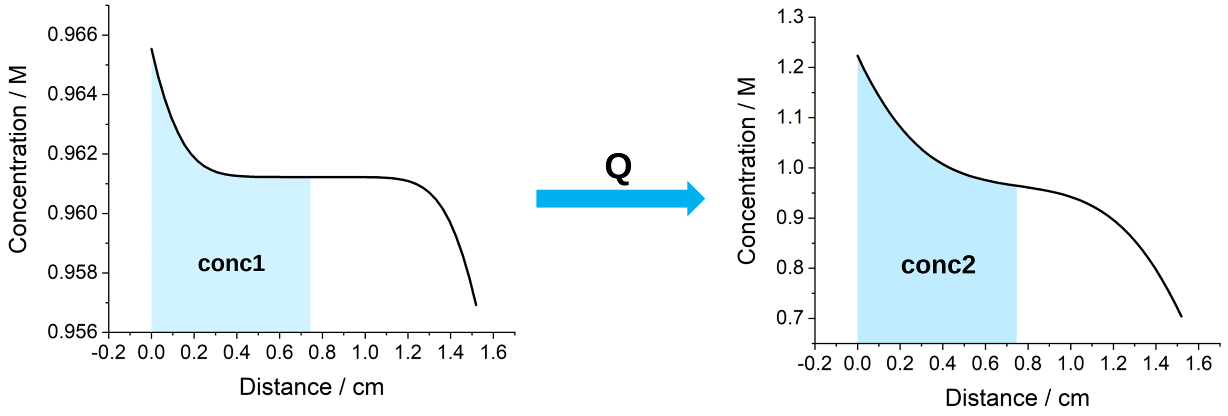

Supplementary Figure 7: *Calculating concentration differences for Hittorf-style transference number measurement, when measuring concentration gradients over time.*

$$t_+^0 = 1 - \frac{\Delta n_{cell}}{n_{charge}} = \frac{F(\Delta conc)A}{Q(1 - c_s V_e)} \tag{24}$$

$$\Delta conc = conc2 - conc1 \tag{25}$$

Where n_{charge} is the number of moles of charge passed, n_{cell} is the molar difference between the two sides of the cell before and after time, t, Q is the charge passed over time, A is the area of the electrode and $conc$ is the concentration change within the blue shaded area. The $(1 - c_s V_e)$ factor accounts for bulk diffusion as described by Monroe et al.[3]

As can be noted from Supplementary Figure 7, $conc1$ does not have to be the initial concentration before current has passed, like that performed for traditional Hittorf measurements.

Supplementary Note 7. Thermodynamic Factor

Supplementary Figure 8a shows how the voltage changed as current passed in the 1m LiFSI in G4 model cell, with Supplementary Figure 8b illustrating how R_{bulk} + R_{ct} stayed quite constant throughout the experiment. This indicated that the increase in voltage was only due to the concentration gradient formation. Supplementary Figure 8c shows how concentration at each cell extreme was progressing throughout the experiment.

For higher concentrations (¿0.5m) ohmic and charge transfer resistances were very steady throughout the experiment. This meant the increasing overpotential was due only to the increasing concentration difference on either side of the cell, with no changes to the interfacial resistance. For lower concentrations (¡0.5 m) PEIS showed an increase in ohmic resistance as the concentration approached zero on the plating side. It would appear that at these concentrations the distribution of Li^{+} ions are non-uniform. It was therefore especially important to perform PEIS throughout the experiment at low concentrations to accurately determine the concentration overpotential.

Fittings of equation 4 (main text) for each concentration were plotted in Supplementary Figure S8d, illustrating the χ_M values extracted from the different gradients.

As mentioned in the main text, χ_M vs. concentration was fitted using $x^{1/2}$ power series, analogous to the Guggenheim extended Debye-Huckel theory.[4] Debye and Huckel developed a theory to describe only how ion association can influence activity values,[5] but with an incomplete picture, multiple variations of their formulation were established.[6, 7] Activity's dependence on the square-root of composition can be noted from the Debye-Huckel equation:

$$log\gamma_{\pm} = -\frac{Az_{+}z_{-}\sqrt{I}}{1 + Ba\sqrt{I}} + CI \tag{26}$$

Where $\gamma_{\pm}$ is the activity coefficient. The fraction on the right-hand side is from classical Debye-Huckel theory, where I is the molar ionic strength, A and B are coefficients informing the extent of association. CI is an additional linear factor that accounts for short-range solvent-ion effects, and the primary basis for extended Debye-Huckel theories. With difficulty fitting χ_M curves with equation 26, most non-aqueous electrolyte used a $x^{1/2}$ power series as suggested by Guggenheim. Our best-fit equation was in Supplementary Figure 4f:

$$\chi_M = 1 + 2.88x^{7/2} - 2.32x + 0.37x^{1/2} \tag{27}$$

For an understanding of the free:bound solvent ratio, at each concentration, a fitting of the solvent peak 780-900 cm^{-1} was performed, shown in Supplementary Figure 8b, illustrating 4 distinct environments. Like Terada et al. we notice at c_{Li}/c_{G4} = 1, which is 3.17 M or 4.44 m, the peak area at 868 cm^{-1} is constant.[8] This suggests all solvent molecules are bound

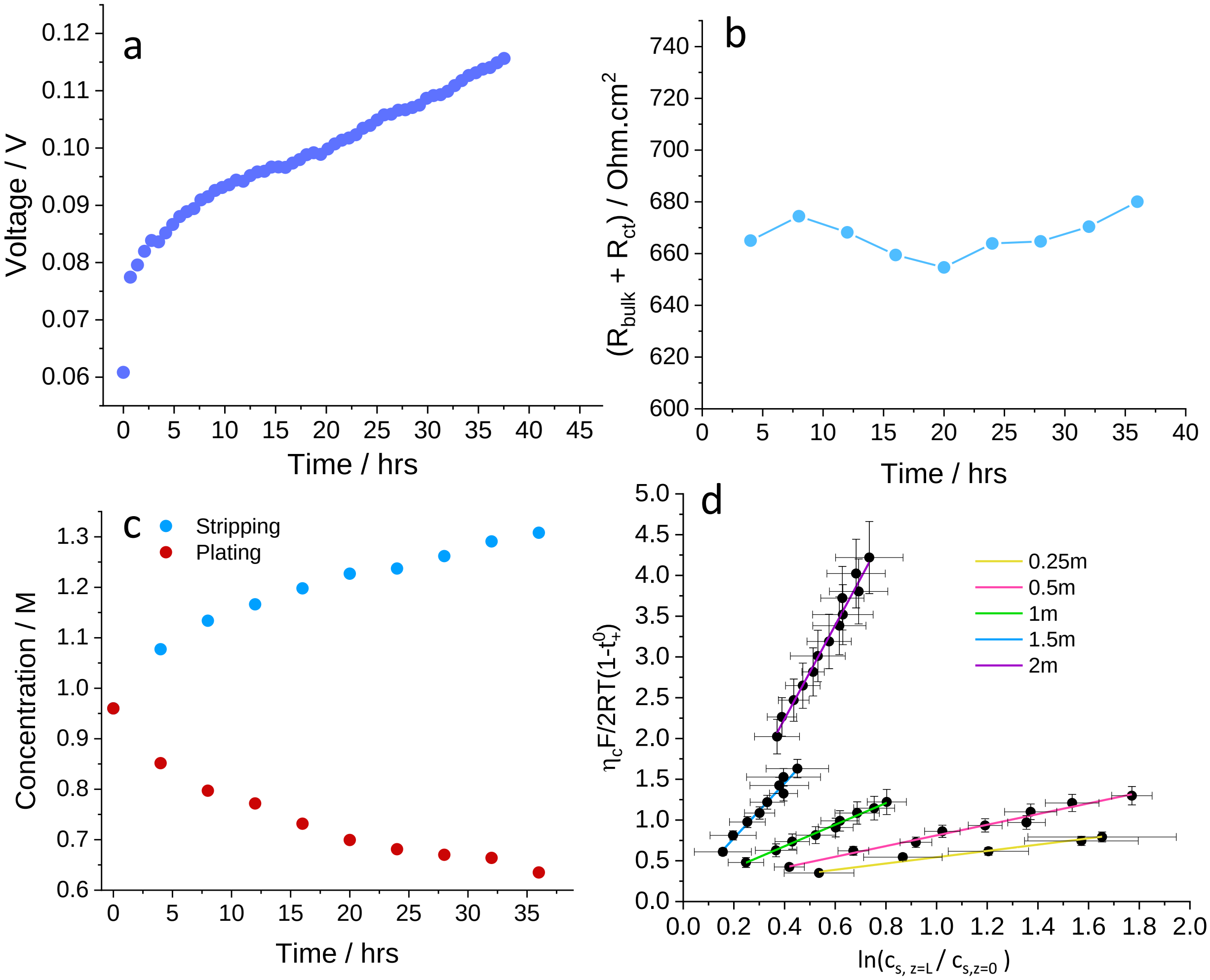

Supplementary Figure 8: *a) Voltage of operando cell with 1m electrolyte, while passing 100 μA cm*$^{-2}$*. The increase in voltage is due to the increasing magnitude of the electrolyte concentration gradient. b)* $R_{bulk} + R_{ct}$ *over time, showing a stable interface with no side reactions c) Concentration at cell plating and stripping electrodes using 1m electrolyte, while passing 100 μA cm*$^{-2}$*. d) Plotting equation 4 (main text) of each electrolyte concentration, illustrating each gradient and ultimately how* χ_M *is varying with concentration.*

from this concentration onwards, meaning there are no free solvent molecules. Like Kottam et al. and Tatara et al., one can normalize this peak, extract the bound solvent and free solvent concentrations, and calculate the solvation number.[9] Table 2 shows how the solvation number is changing with concentration. Clearly, as concentration increases the solvation number decreases due to the availability of solvent molecules with increasing concentration. The increasing amount of bound solvent as the $[Li(G4)]^+$ complex would lead to an increase in χ_M.

Supplementary Table 2: *Ratio of free:bound solvent, with solvation number*

$\text{Concentration}_{molality}$	$\text{Concentration}_{molarity}$	$[G4]_{bound}$	$[G4]_{free}$	Solvation Number
4.44	3.17	3.171	0.000	1.000
4	3	3.227	0.143	1.076
3	2.44	2.668	0.998	1.093
2	1.75	2.196	1.765	1.255
1	0.95	1.257	3.011	1.323
0.5	0.49	0.659	3.771	1.318

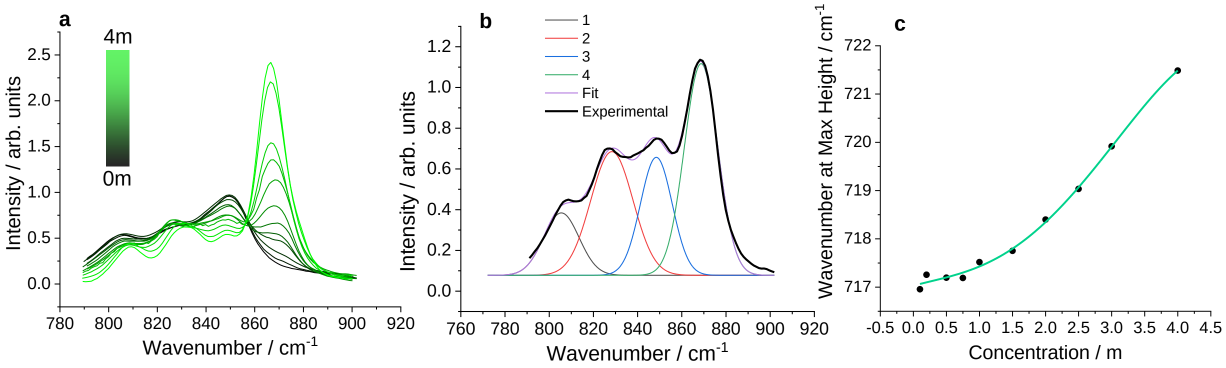

Supplementary Figure 9: *a) Evolution of 868 cm^{-1} peak with concentration b) Fitting of 2m 780-900 cm^{-1} peak, showing 4 distinct environments c) Wavenumber at max height of 717 cm^{-1}, showing a dramatic shift from ~1 m*

Supplementary Note 8. Stefan-Maxwell Diffusion Coefficients

Most articles that rigorously characterise electrolytes base their characterisation on concentrated-solution theory, outlined by Newman and Thomas-Alyea.[3, 10, 11]

Using Stefan-Maxwell theory it is the electrochemical potential gradient which is the driving force that induces diffusion. As stated by Rehfeldt et al., Stefan and Maxwell assume an equilibrium of thermodynamic interaction between the species and molecular friction; if out of equilibrium this leads to flux by diffusion.[12]

D_{app}, derived from Fick's Laws of diffusion, can be defined using concentrated-solution theory [1, 10] by equation 28.

$$D_{app} = \mathfrak{D}\frac{c_T}{c_0}\chi_M(1 - c_s V_s) \tag{28}$$

Where:

$$\mathfrak{D} = \frac{2D_{0+}D_{0-}}{z_+D_{0+} - z_-D_{0-}} \tag{29}$$

$\mathfrak{D}$ is the thermodynamic diffusion coefficient, where $\mathfrak{D}_{0+}$ and $\mathfrak{D}_{0-}$ are the Stefan-Maxwell intermolecular diffusion coefficients of the anion or cation and their interaction with the solvent, depending on the subscript. $\mathfrak{D}$ is the proportionality constant that is influenced by electrochemical potential gradients (thermodynamic driving force) instead of the observed concentration gradients. Where c_T is the total concentration and c_0 is the concentration of the solvent.

Also, individual Stefan-Maxwell diffusion coefficients based on ion-ion and ion-solvent interactions can be calculated:

$$\mathfrak{D}_{0+} = \frac{-z_-}{z_+ - z_-}\frac{\mathfrak{D}}{1 - t_+^0} \tag{30}$$

$$\mathfrak{D}_{0-} = \frac{z_+}{z_+ - z_-}\frac{\mathfrak{D}}{t_+^0} \tag{31}$$

$$\mathfrak{D}_{+-} = \left(\frac{z_+z_-c_TF^2}{\kappa RT} - \frac{z_+ - z_-}{z_+\nu_+}\frac{c_0t_+^0(1 - t_+^0)}{c\mathfrak{D}}\right)^{-1} \tag{32}$$

For a rigorous discussion and derivation of these equations, readers are referred to original work by Newman and Thomas-Alyea.[1, 13]

Supplementary Discussion 1. Cell Simulations

By measuring parameters D_{app}, t_+^0, κ and χ_M one can implement these values into a Doyle-Fuller-Newman (DFN) model and simulate symmetric and full cell performance. We simulated cells using the Batteries and Fuel Cells Module in COMSOL Multiphysics 5.5.

Supplementary Figures 9a-c simulate a symmetric cell with a plating and stripping electrode, similar to the cell described in this study, except the interelectrode distances are at 15 µm, 50 µm and 100 µm, and the current density applied is 5 mA cm^{-2}. In this example, 1m LiFSI G4 was used, with the measured properties from the *operando* Raman microspectroscopy experiment. Indeed, the measured parameters in this study are independent of current density and interelectrode distance, but it illustrates the extent and speed of concentration gradient formation at more realistic LMB geometries.

Supplementary Figures 9d-f simulate a full LIB cell geometry using porous graphite and porous lithium manganese oxide (LMO) as the negative and positive electrode respectively. Full cell parameters are described in the table below. Supplementary Figure 9d shows a galvanostatic discharge curve at a 1C C-rate, illustrating how electrolyte concentration, transport and thermodynamic properties can influence the shape of the curve, and ultimately overall cell performance. Interestingly, it is 1m and 0.5m that performs best, showing the least overpotential. Supplementary Figure 9f shows how the concentration gradient of the electrolyte progresses over 1h. Supplementary Figure 9e highlights even further how electrolyte composition can influence cell performance, when increasing the C-rate to 4C. The biggest influencer on final state-of-charge (SOC) is the bulk concentration, with 0.25m and 0.5m performing particularly poorly. Supplementary Figure 10a-b shows the difference in electrolyte concentration distribution for the 0.5m electrolyte discharged at 1C and 4C respectively. Due to the low initial concentration throughout the cell and a high C-rate causing large concentration gradients, the concentration in the cathode drops to zero in much of the electrode. This leads to a low final average SOC at a 3V cut-off.

But, again it is 1 m that achieves the best results, showing the least overpotential and highest final capacity at a 3 V cut-off.

A 1D model was used to simulate each setup. For the symmetric cell, distances of 15 µm, 50 µm and 100 µm between two nodes were used. For the full cell, graphite and LMO were implemented, Li_xC_6—electrolyte—$Li_yMn_2O_4$. The graphite thickness was 100 µm, the electrolyte 50 µm and LMO was 185 µm.

DFN modelling uses concentrated solution theory and porous electrode theory, developed by Newman.[1] The equations related to these theories are implemented in the Fuel Cells and Batteries Module in COMSOL Multiphysics 5.5. In the module are parameters for each porous electrode including electric conductivity and lithium diffusion coefficient. See table 3 for full details. For a detailed description of the model, readers can refer to work by Newman.[1, 14] Furthermore,

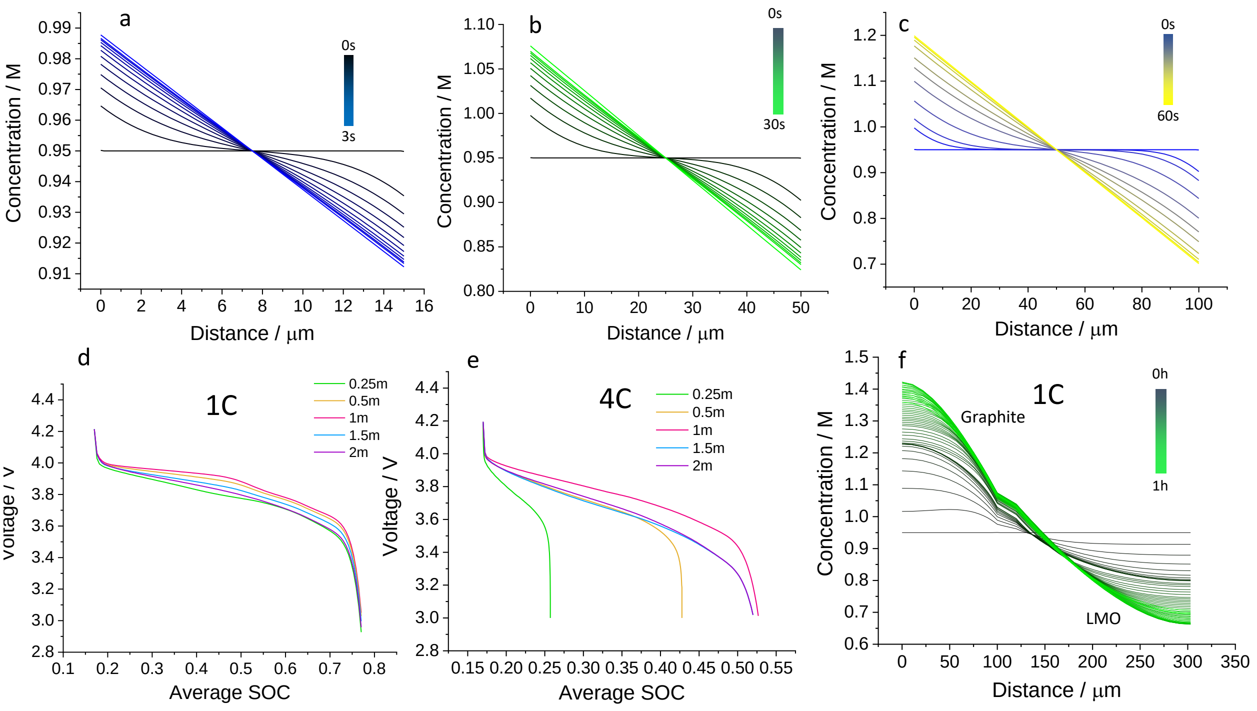

Supplementary Figure 10: *a-c) Simulation of the 1 m LiFSI in G4 electrolyte concentration gradient forming in a lithium symmetric cell at a) 15 µm b) 50 µm c) 100 µm, at 5 mA cm^{-2}. d-f) Simulation of a full LIB cell, with porous graphite and porous LMO as the negative and positive electrodes respectively. d) Discharge curve simulation at 1C, illustrating that the 1m concentration performed with the least overpotential. e) Discharge curve simulation at 4C, showing cell performance with a high dependence on concentration with 0.25 m and 0.5 m performing poorly. 1 m performed at the least overpotential and highest final SOC at 3 V cut-off. f) Electrolyte concentration profile at 1C of 1m initial concentration.*

others in the field have utilised this model to understand how parameters such as transference number may affect cell performance.[15]

Symmetric Cell

The DFN model combines concentrated solution theory to account for electrolyte flux in non-ideal solutions, and porous electrode theory to understand behaviour in porous microstructures.

Molar flux in a binary electrolyte is governed by:

$$N_i = -D_{app}\nabla c_i + \frac{i_i t_+^0}{F} \tag{33}$$

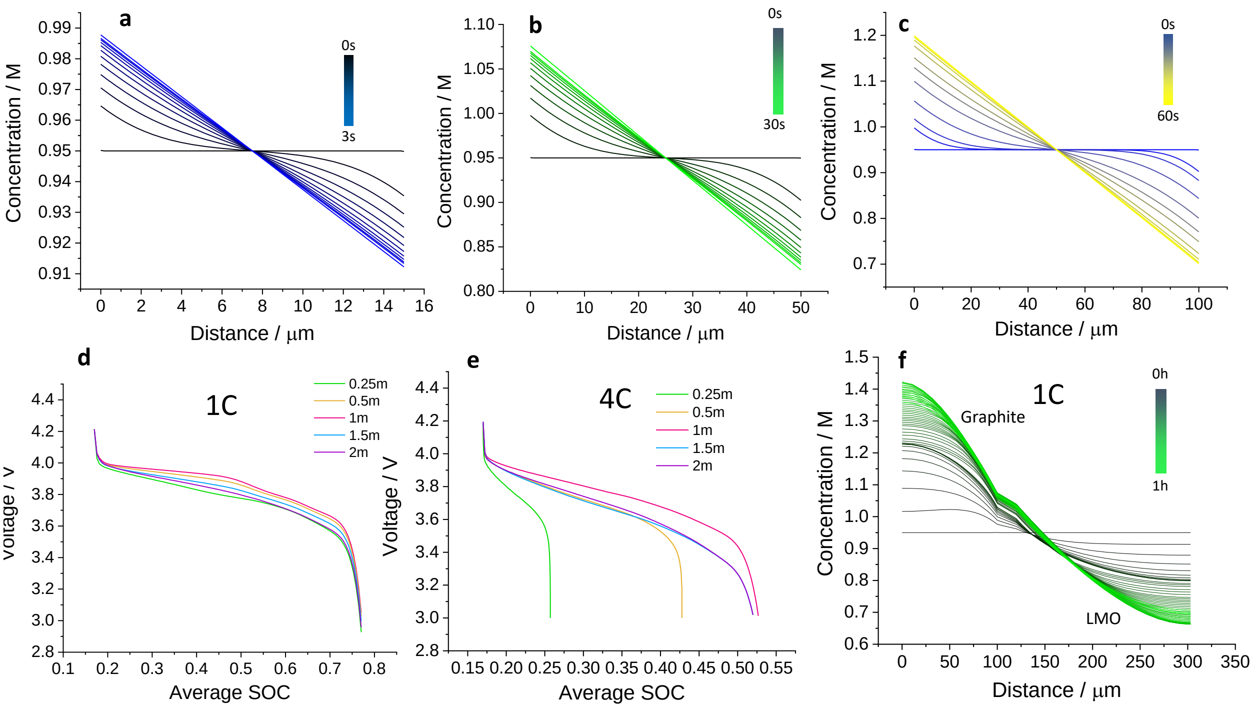

Supplementary Figure 11: *a) Electrolyte concentration profile at 1 C with 0.5 m initial concentration. b) Electrolyte concentration profile at 4 C with 0.5 m initial concentration.*

Where N_i is the molar flux of i, F is the Faraday constant and i_i is the current density of i and c_i. To know current density, one needs to understand how electrolyte potential is varying in solution:

$$i_i = -\kappa \nabla \Phi_i + \frac{2\kappa RT}{F} \chi_M (1 - t_+^0) \nabla \ln c_i \tag{34}$$

Where Φ_i is measured with a lithium reference electrode, T is the temperature, R is the ideal gas constant.

In short, equations 33 and 35 describe how electrolyte potential and concentration vary in an electrolyte. Then, one needs to set the boundary conditions. At one boundary (x=0), the potential is set to zero, and the other (x=L) includes the flux of lithium ions:

$$N_+ = \frac{I}{F} \tag{35}$$

An applied current of 5 mA cm^{-2} was used in our simulations.

To measure how current at the interface influences surface overpotential, the Butler-Volmer equation was used:

$$i_{loc} = i_0 \left(\exp \frac{\alpha_a F \eta}{RT} - \exp \frac{\alpha_c F \eta}{RT} \right) \tag{36}$$

i_{loc} is the local current density at the interface, η is the local activation overpotential, α_i is the anodic and cathodic transfer coefficients and i_0 is the exchange current density. i_0 was assumed to be 1 A/m^2.

Full Cell

Full cell simulations utilised the equations above to describe electrolyte concentration and potential, and reaction kinetics at the interface. But further to this, porous electrode theory was used.

For full cell simulations the porous electrode consisted of spherical active material particles and LiFSI in G4 electrolyte in the void space. The electric current in the active material particles was defined by Ohm's Law:

$$i = -\sigma_s \nabla \Phi_s \tag{37}$$

Where σ_s is the electric conductivity of the material.

With the full cell consisting of insertion electrodes, Li^+ diffusion was defined by Fick's Law:

$$N_s = -D_s \nabla c_s \tag{38}$$

$$\frac{\partial c_s}{\partial t} = \nabla (D_s \nabla c_s) \tag{39}$$

Where D_s is the diffusion coefficient of Li^+ in the material, and c_s is the concentration of lithium in the solid phase.

The interfacial kinetics were again defined by equation 36 with i_0 defined as:

$$i_0 = Fk(c_s^{max} - c_s)^{\alpha_a} (c_s)^{\alpha_c} (c_l)^{\alpha_a} \tag{40}$$

Where k is the rate constant and c_s is the solid phase lithium concentration.

The current density was specified at the cathode/current collector interface.

Supplementary Table 3: *COMSOL Simulation Parameters*

Parameter	Value
Temperature	298.15 K
Electrolyte thickness	50 μm
Graphite thickness	100 μm
LMO thickness	185 μm
Positive electrode LMO volume fraction	0.273
Positive electrode electrolyte volume fraction	0.654
Negative electrode graphite volume fraction	0.471
Negative electrode electrolyte volume fraction	0.503
Anodic/cathodic rate coefficients for each electrode	0.5
Reference exchange current density	100 A/m^2
Electric conductivity graphite	100 S/m
Electric conductivity LMO	3.8 S/m
Initial lithium concentration in graphite	17644 mol/m^3
Initial lithium concentration in LMO	3886 mol/m^3
Radius of graphite particles	12.5E-6 m
Radius of LMO particles	8E-6 m
Diffusion of lithium in graphite	3.10E-14 m^2/s
Diffusion of lithium in LMO	1.05E-14 m^2/s

Electrolyte flux and potential were defined by equations 33 and 35. Measured values of D_{app}, t_+^0, κ and χ_M were used in the simulations.

Supplementary References

1. Newman, J. & Thomas-Alyea, K. E. *Electrochemical Systems* 3rd (Wiley & Sons, 2004).
2. Bard, A. J. & Faulkner, L. R. *Electrochemical Methods* 2nd ed. (Wiley & Sons, 2001).
3. Hou, T. & Monroe, C. W. Composition-dependent thermodynamic and mass-transport characterization of lithium hexafluorophosphate in propylene carbonate. *Electrochimica Acta* **332,** 135085 (2020).
4. Guggenheim, E. *Thermodynamics: An Advanced Treatment for Chemists and Physicists* 5th (North-Holland Publishing Company, 1950).
5. Koryta, J., Dvorak, J. & Kavan, L. *Principles of Electrochemistry* 2nd (Wiley & Sons, 1987).
6. Pan, C.-F. A simplified form of Stokes-Robinson equation. *Journal of Chemical Information and Modeling* **54,** 1689–1699 (1976).
7. Nesbitt, H. W. The stokes and robinson hydration theory: A modification with application to concentrated electrolyte solutions. *Journal of Solution Chemistry* **11,** 415–422 (1982).
8. Terada, S., Ikeda, K., Ueno, K., Dokko, K. & Watanabe, M. Liquid structures and transport properties of lithium bis(fluorosulfonyl)amide/glyme solvate ionic liquids for lithium batteries. *Australian Journal of Chemistry* **72,** 70–80 (2019).
9. Kottam, P. K., Dongmo, S., Wohlfahrt-Mehrens, M. & Marinaro, M. Effect of salt concentration, solvent donor number and coordination structure on the variation of the Li/Li+ potential in aprotic electrolytes. *Energies* **13** (2020).
10. Nyman, A., Behm, M. & Lindbergh, G. Electrochemical characterisation and modelling of the mass transport phenomena in $LiPF_6$-EC-EMC electrolyte. *Electrochimica Acta* **53,** 6356–6365 (2008).
11. Ehrl, A., Landesfeind, J., Wall, W. A. & Gasteiger, H. A. Determination of Transport Parameters in Liquid Binary Lithium Ion Battery Electrolytes: Part I. Diffusion Coefficient. *Journal of The Electrochemical Society* **164,** A826–A836 (2017).
12. Rehfeldt, S. & Stichlmair, J. Measurement and calculation of multicomponent diffusion coefficients in liquids. *Fluid Phase Equilibria* **256,** 99–104 (2007).
13. Newman, J., Bennion, D. & Tobias, C. W. Mass Transfer in Concentrated Binary Electrolytes. *Berichte der Bunsengesellschaft für physikalische Chemie* **69,** 608–612 (1965).
14. Doyle, M., Fuller, T. F. & Newman, J. The importance of the lithium ion transference number in lithium/polymer cells. *Electrochimica Acta* **39,** 2073–2081 (1994).
15. Diederichsen, K. M., McShane, E. J. & McCloskey, B. D. The Most Promising Routes to a High Li^+ Transference Number Electrolyte for Lithium Ion Batteries. *ACS Energy Letters* **2,** 2563–2575 (2017).
